# Invisalign treatment from the patient perspective: A Twitter content analyses

**DOI:** 10.4317/jced.57835

**Published:** 2021-04-01

**Authors:** Milagros Adobes-Martin, Maria-Luisa Montoya-Morcillo, Angel Zhou-Wu, Daniele Garcovich

**Affiliations:** 1 Professor, Department of Orthodontics, Universidad Europea de Valencia. Spain. Department of Pediatric dentistry, Dental School, University of Valencia, Valencia, Spain; 2 Resident, Department Orthodontics, Universidad Europea de Valencia. Spain; 3 Professor, Department Orthodontics, Universidad Europea de Valencia. Spain; 4 Professor, Department of Orthodontics, Universidad Europea de Valencia. Spain

## Abstract

**Background:**

Understanding patient perspective is a key factor in improving treatment satisfaction. The aim of the present study is to qualitatively describe the content of Twitter posts related to the treatment with Invisalign in order to get a better understanding of patient experience.

**Material and Methods:**

Tweets were prospectively collected during a period of four weeks from public available posts on Twitter using Awario™ a bespoke social media monitoring tool. After applying the pertinent inclusion and exclusion criteria the selected posts were analysed by three investigators using thematic analysis. Specific themes and subthemes were developed.

**Results:**

A total of 1564 tweets were analysed; three mayor themes were identified: Pre-treatment related, Treatment related and Patient/clinician relationship. Pre-treatment posts were mainly positive and underlined patients’ expectations, while in the treatment phase an almost equal number of positive and negative posts were found. The positive post were about the satisfaction with treatment and the improvement of self-esteem. The negative ones were related to pain, compliance, the impact on diet and pronunciation.

**Conclusions:**

The present study provides a better understanding of patients’ experience during clear aligners treatment. Increasing the awareness of the clinicians can improve their ability to face problems related to the orthodontic treatment and to provide to their patients better professional advices and counselling.

** Key words:**Social media, twitter, clear aligners, Invisalign, orthodontics.

## Introduction

Patient doctor relationship changed dramatically in the last decades and dental practices have experienced long ago the transition from doctor-centred to patient-centred dental care. In this new setting the issue of patient satisfaction gained more and more importance as highlighted by the number of PubMed articles featuring patient satisfaction as a key-word that increased from 304 in 1990 to 8202 in 1999. In the United States new regulations require that physician performance have to be assessed and graded in both objective and subjective ways and patient satisfaction has been established as a key component of physician rankings and reimbursement (1). Patient perspectives are so important that high impact journals as the British Medical Journal BMJ have launched the Patient and Public Partnership strategy, designed to promote co-production of the journal content and enhance the global debate on patient and public involvement in healthcare and health research (2). The authors submitting research papers have to declare if and how they involved the patients or the public in their work. Papers are also sent for review by patients and public reviewers, as well as to peer reviewers.

Many factors are involved in patient satisfaction, that can be considered a blend of the patient’s beliefs, the perceived impact of treatment on the quality of life and the perceived quality of service provided by the dental team. The standard way to asses patients perspective and satisfaction relies on surveys and questionnaires that can present a risk of bias due to patients reluctance to share their experience or their will to please the clinician (3). In this perspective, Twitter, a microblogging platform that allows the users to send, share and read short messages up to 140 characters, represents the ideal place for patients to express in real time their straightforward opinion. Twitter, since its establishment in 2006, reached as much as 350 millions users in 2019

, and involves according to a recent survey about a third of the orthodontic patients (4) being the second most used social media in this group of people (5). This microblogging platform is a recognized mean of dissemination for the health service stakeholders and is included as a core source in Altmetric, a novel alternative metric for research outputs (6). The aim of the present study is to qualitatively describe the content of Twitter posts related to the treatment with Invisalign in order to get a better understanding of patient experience along the treatment and anticipate the problems the patient could face in order to deliver a better standard of care.

## Material and Methods

Tweets were prospectively collected from public available posts on Twitter (www.twitter.com) using a free version of a bespoke social media monitoring tool (https://awario.com). Awario™ property of SEO software is a social media monitoring software that tracks every corner of the web for mentions related the selected keywords in real time. The software tracks the growth in the number of mentions and their collective reach and is able to sort mentions by positive, negative, and neutral with a so called sentiment analysis. The search was limited to original English language tweets. Tweets containing the keywords “Invisalign” OR “Invisalign treatment” were collected over a total four weeks: two weeks from October 1st to October 15th, 2019 and two weeks from January 25th to February 8th, 2020. The harvested tweets were exported to an excel spreadsheet (version 14.2.0, Microsoft, Redmond, WA, USA). The exported data included the tweet, date and time of posting, and the number of followers. Tweets were then selected according to the following inclusion and exclusion criteria. Inclusion criteria: posts related to Invisalign treatment. Exclusion criteria: unclear content, not in English, irrelevant to Invisalign, duplicates, advertisement or promotional posts and posts involving statements that did not include content that could be used to make inferences about how people felt about their appliance. The screening was performed simultaneously but not independently but two of the authors (and). If a link was provided in the tweet, it was investigated to better understand the content. Each tweet was categorized according to its content; in certain scenarios, some tweets were categorized under several themes. The tweets were then classified by themes and subthemes ( Table 1). The tweets were categorized under positive, negative or neutral feelings, according to the feeling expressed by the tweet author. The same tweet could be categorized under different themes if a feeling was expressed and the reason behind it. I.e.: I am angry because I can’t eat my favourite food (negative feeling- impact on diet quality). To classify the tweet author into patient or professional, the public profile was checked if required. Even if Awario™ performs a sentiment analysis, tweets were analysed and coded manually. Due to their shortness, the use of slang, abbreviations and ironic unconventional written expressions, manual coding can result in a better stratification of the selected tweets, since humans can detect the nuances of written expression better than artificial algorithms (7). The data were then categorized under different themes and subthemes through a thematic analysis based on the guidelines of Braun and Clarke (8). Thematic analysis involve the repeated reading of all of the tweets, marking ideas and notes in relation to Invisalign® treatment. Three investigators (and), with different clinical backgrounds, independently and not simultaneously read and analysed the selected tweets. The investigators were blinded to the identity of the Twitter users and to the outcome of the others investigators in the team during content analysis. After sharing the work the investigators grouped the tweets together into topics and then reviewed and refined until meaningful and distinct main themes and subthemes were developed. Areas of disagreement between investigators were resolved by consensus.

**Table 1:**
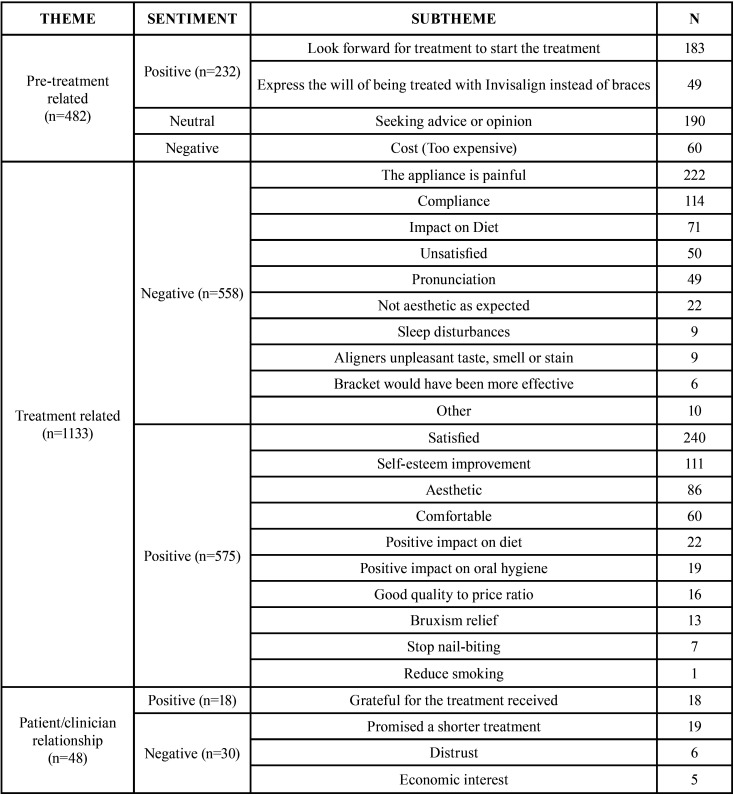
Distribution of tweets in each theme and subtheme. Number of post related to every subtheme is presented (N).

## Results

**Figure 1:**
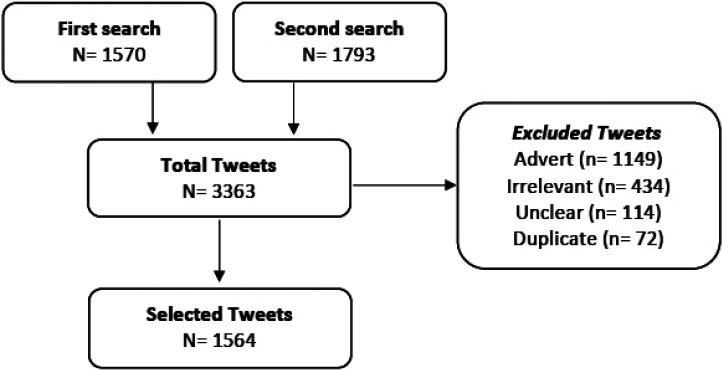
Flow diagram of the search process.

Along the four weeks period, a total of 3363 tweets were tracked by the software and manually assessed. Out of the total pool, 1149 posts were related to adverts, mostly of dental clinics offering Invisalign treatment. The flow of tweets selection is displayed in Figure 1. The selected 1564 tweets were grouped in three mayor categories: Pre-treatment related, Treatment related and Patient/clinician relationship. The pre-treatment related tweets were mainly of patients seeking advice or opinion of their online peers or expressing their will or impatience to start the treatment ( Table 1). There was a high interest in the pre-treatment phase and a 11,6% of the tweets were specifically reporting a preference toward Invisalign as a treatment modality. Positive tweets outweigh the negative ones in the pre-treatment phase. Negative tweets in this phase were mostly related to the treatment cost.

Analysing the treatment related tweets it is possible to appreciate how they are almost equally divided between positive and negative ones. The negative ones were mainly related to the pain associated with treatment. Only few of them specified that pain was related to the aligner change (n=4) or the attachments (n=3). Compliance related problems were reported in 114 posts mentioning aligners loss, a suboptimal wearing time or not wearing the elastics. Matter of concern was also the impact on diet and on pronunciation, especially lisping reported in 9 out of 40 tweets. When reporting that the appliance was not as aesthetic as expected 8 out of 22 tweets pointed out the negative impact of attachments on overall aesthetic. On the other hand most of the positive tweets were related to patient satisfaction with the appliance results and the impact of their new appearance on self-esteem. The appliance was deemed comforTable and aesthetic in a large number of posts, while many posts highlighted how the appliance was actually helping to regularize the diet (patients avoided snacking between meals), enhancing the oral hygiene standards and limiting harmful habits (clenching, nail-biting and smoking) ( Table 1).

On the patient/clinician side, the number of positive posts from grateful patients is counterbalanced by the negative ones pointing out to the excessive treatment length in respect of what planned at the beginning. A synopsis of the representative tweets illustrating each of the identified subthemes is displayed in  Table 2.

**Table 2:**
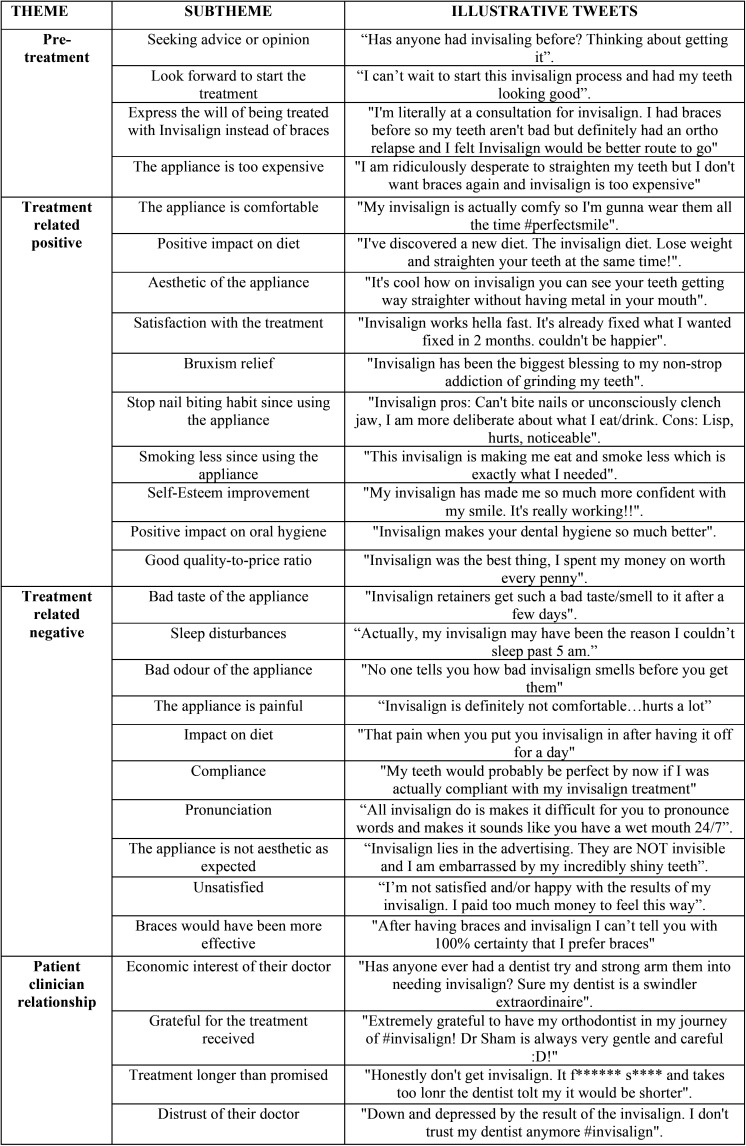
Representative tweets illustrating each of the identified subthemes.

## Discussion

According to our best knowledge only one author has previously published a research about Invisalign perception on Twitter, based on a software based sentiment analyses (9). Computerized tools allow to manage a high number of data but with less attention to details and the trending topics are usually identified by keywords tracking. Our method allow to manage a lower number of posts but permits a deeper analyses. Al-Moghrabi *et al.* in a Twitter investigation on retainer included 660 tweets (10), Watts *et al*. included 689 tweets in a Twitter analyses on Orthodontics and Orthognatic surgery (11), while Graf *et al.* included as little as 156 Tweets in a research about orthodontics and social media (12). Compared to these pervious reports, using manual coding, our study included a much larger sample size.

Based on the number of tweets, tracked by the software it seems that Twitter is a platform chosen by a significant number of orthodontic patients to share experiences related to their Invisalign treatment. The search result highlights how it is also a preferred site for orthodontic practices to advert their treatments. Our results are consistent with the ones of other authors who found that 33% of the tweets about Invisalign were related with advertisements (9).

In the pre-treatment phase the clear prevalence of positive contributions underlines how the appliance is appealing to the general public, probably demonstrating the positive outcome of the brand building campaigns carried out by Align Technology (San Jose, Calif), the owner of its trademark, along the past decades. In this phase a relevant number of posts complained about the cost showing how the high price of the appliance is not a facilitator for the patient to choose this treatment alternative, being this finding similar to what reported by other authors (13).

Social networking is a key feature of the human being that learnt, through time and evolution, that living in a community where sharing knowledge, experiences, and skills, presented a definite advantage. The communication network that was once confined to family, friends, and neighbours with the advent of social media experienced an enormous expansion and become almost boundless. Social networking favouring contacts and giving the opportunity to influence others in many ways, have definitely revolution the way people relate with one another (14). In this scenario is tremendously important for the health care professionals to know what´s going on social media (15). In a previous investigation Noll *et al.* reported that a 62% of the collected tweets had a positive component while the 38% had a negative one. In our sample the prevalence of negative tweets is similar, but higher in the treatment-phase. This difference can be explained with the different methods used for sentiment analyses. Noll *et al.* used a software based one while we did it manually. A manual detailed selection of individual comments is deemed to be more accurate than a software based one according to various authors (10,11,16). A high percentage of negative tweets can be a surprising finding for a treatment strongly marketed by the owning company as a treatment offering an improved patient experience over traditional braces, in terms of comfort, treatment time, force level, overall aesthetic and an improved quality of life. Probably the difference between the treatment real impact and the expected or advertised one can increase the perceived discomfort once in treatment.

Posted tweets can be considered a type of word of mouth WOM, which has come to be called in these cases electronic word of mouth or eWOM. Health care providers as every other service provider should pay attention to customers’ opinions, especially due to the enormous dissemination potential of eWOM. It is of considerable interest to underline that according to current evidence the eWOM is more powerful than any communication or marketing campaign carried out by companies (17). Most authors in the field of business management have pointed out how comments are posted when a customer has either an extremely good or an extremely bad experience. The most satisfied or unsatisfied customers are the ones who are posting more commentaries, while the vast majority of customers who had a positive but more neutral experience, will be more passive in terms of online reviews (18). In this perspective the clinician should be prepared to face the events that are related to the most negative posts thus avoiding negative experience. According to our results and what reported by other authors the highest number of negative posts was related to pain (9,13). Although pain level seems to be less for Invisalign treated patients when compared to patients treated with edgewise (19) or self-ligating brackets (20) some authors reported how aligners distortion can be cause of increased pain sensation (21). Avoiding tray distortion and change the new aligner only when the proper fit is ensured, can provide a smoother treatment. Compliance related issues were also frequent in our sample as reported by other authors in studies on removable appliances (10). Compliance related problems will probably increase along with the increase of aligner treatment targeting children and adolescents who have demonstrated worst compliance behaviour (22). Therefore, the clinician should try to adopt all the possible strategies to increase compliance as asking for compliance indicators in the aligners and implementing systems of e-mail, text message or application based reminders that can enhance patients’ adherence to treatment.

Traditional research tools used to assess patients’ perspectives, such as surveys or questionnaires have the drawback of reducing the themes asking specific questions and so restricting the emergence of new patient based perspectives derived from their very unique experience of the treatment. Face-to-face interviews can theoretically overcome the shortcomings of questionnaire but have some limitations associated with the reluctance that could some patients have in sharing experiences and perspectives in an uninhibited manner due to the interpersonal contact (11). Twitter instead allows to collect the unvarnished opinions of patients sheltered by their fairly anonymous profiles and nicknames and is therefore a preferred place to know about their true own experience. One of the limitation of the present study is that through the platform the demographic patients’ data are not accessible, not allowing the interpretation of data in relation to the sex and age cohort. Moreover limiting the analyses to tweets in English language have restricted the study reach to the English speaking countries, making difficult to extrapolate the result to other regions. The present study was limited to a relatively short period of data tracking, but the use of a longer study period could have generated an overwhelmingly volume of data, probably too large to manage and analyse.

## Conclusions

According to the findings of the present study the treatment with clear aligners, especially in the pre-treatment phase, is related to high expectations over aesthetic comfort and performance probably due to the strong marketing that surrounds this treatment. In the treatment phase, negative experiences arise and are widely shared on Twitter. The knowledge of patient perspectives can help clinicians involved in the treatment with clear aligners to deal properly with the problems that can turn up during the treatment process as the ones related to pain, compliance, diet or pronunciation. Moreover the orthodontists aware of the most important problems related to this type of treatment can offer to their patients better professional advices and counselling, increasing patient satisfaction.
